# Educational attainment in survivors of childhood cancer in Denmark, Finland, and Sweden

**DOI:** 10.1038/s41416-023-02499-1

**Published:** 2023-11-22

**Authors:** Hanna Mogensen, Giorgio Tettamanti, Line Elmerdahl Frederiksen, Mats Talbäck, Juho Härkonen, Karin Modig, Camilla Pedersen, Anja Krøyer, Elli Hirvonen, Anniina Kyrönlahti, Mats Heyman, Anna Sällfors Holmqvist, Henrik Hasle, Laura Madanat-Harjuoja, Nea Malila, Jeanette Falck Winther, Friederike Erdmann, Maria Feychting

**Affiliations:** 1 Unit of Epidemiology, Institute of Environmental Medicine, Karolinska Institutet, Stockholm, Sweden; 2 Childhood Cancer Research Group, Danish Cancer Institute, Copenhagen, Denmark; 3 Department of Political and Social Sciences, European University Institute, Florence, Italy; 4 Department of Sociology, Stockholm University, Stockholm, Sweden; 5 Finnish Cancer Registry, Cancer Society of Finland, Helsinki, Finland; 6 New children’s Hospital, University of Helsinki and Helsinki University Hospital, Helsinki, Finland; 7 Childhood Cancer Research Unit, Department of Women’s and Children’s Health, Karolinska Institutet and Karolinska University Hospital, Stockholm, Sweden; 8 Department of Clinical Sciences, Lund University, Childhood Cancer Center, Skåne University Hospital, Lund, Sweden; 9 Department of pediatric and adolescent medicine, Aarhus University Hospital, Aarhus, Denmark; 10 Dana-Farber/Boston Children’s Cancer and Blood Disorders Center, Boston, MA USA; 11 Department of Clinical Medicine, Faculty of Health, Aarhus University and Aarhus University Hospital, Aarhus, Denmark; 12 Research group Aetiology and Inequalities in Childhood Cancer, Division of Childhood Cancer Epidemiology, Institute of Medical Biostatistics, Epidemiology and Informatics (IMBEI), University Medical Center of the Johannes Gutenberg University Mainz, Mainz, Germany; 13 Department of Prevention and Evaluation, Leibniz Institute for Prevention Research and Epidemiology – BIPS, Bremen, Germany

**Keywords:** Paediatric cancer, Cancer epidemiology

## Abstract

**Background:**

Survivors of childhood cancer may face difficulties at school. We investigated whether childhood cancer affects attainment of upper secondary education, in a register-based cohort study from Denmark, Finland, and Sweden, where we limit bias from selection and participation.

**Methods:**

From the national cancer registers, we identified all long-term survivors of childhood cancer diagnosed aged 0–14 years in 1971–2005 (*n* = 7629), compared them to matched population comparisons (*n* = 35,411) and siblings (*n* = 6114), using odds ratios (OR) and 95% confidence intervals (CI).

**Results:**

Overall, 6127 survivors (80%) had attained upper secondary education by age 25, compared to 84% among comparison groups. Elevated OR for not attaining this level were mainly confined to survivors of central nervous system (CNS) tumours (OR_Surv_PopComp_2.05, 95%CI: 1.83–2.29). Other risk groups were survivors who had spent more time in hospital around cancer diagnosis and those who had hospital contacts in early adulthood, particularly psychiatric. Survivors of all cancer types were less likely to have attained upper secondary education without delay.

**Conclusions:**

Although survivors of childhood cancer experienced delays in their education, many had caught up by age 25. Except for survivors of CNS tumours, survivors attained upper secondary education to almost the same extent as their peers.

## Introduction

Advances in the diagnosis and treatment of childhood cancer have led to remarkable improvements in survival and a steadily increasing population of survivors [1, 2]. Attention has therefore been paid to better understand the somatic late effects and adverse socioeconomic consequences of a childhood cancer diagnosis [3, 4]. Educational attainment, such as completion of upper secondary education, are important milestones for future work-life opportunities. Survivors of childhood cancer may experience educational difficulties due to absence from school while undergoing treatment, the disease itself, or the toxicity of cancer treatment, which can affect cognition [5]. In previous studies, survivors of central nervous system (CNS) tumours and those treated with CNS-directed therapy were shown consistently to have lower educational attainment than their peers [4–7]. The picture is less conclusive for survivors of other cancer types, previous studies having reported that survivors of this heterogeneous group of malignancies have worse, equal, or better educational outcomes than comparison groups [4–7]. A recent review of the literature, including guidelines for surveillance, concluded that childhood and young adult cancer survivors are at increased risk for lower educational achievement overall, but the evidence level was graded as very low [8]. Most previous studies had methodological limitations such as use of self-reported outcomes from questionnaires, risk of selection bias due to non-participation, limited follow-up time, and assessment of educational attainment at only one time. The existing literature does not, therefore, clearly show whether survivors experience only delays in educational attainment [9], or whether they continue to lag behind their peers in adulthood.

In many previous studies, subgroup analyses were lacking owing to the rarity of childhood cancer. Diagnosis-specific analyses are, however, clinically relevant and important for potential interventions. The risk factors for lower educational attainment include not only the diagnostic group [4–7] but also the age at diagnosis [4, 10]. The severity of disease and somatic and psychiatric late effects [3, 11] have rarely been considered in previous work on educational attainment, although these factors can substantially affect both school attendance and the ability to benefit from teaching. Parental education is another factor that is likely to influence educational attainment also among childhood cancer survivors [9, 12, 13], and may confound the association. Moreover, highly educated parents may be in a better position to support their children during and after cancer treatment and thus compensate for the negative effects of childhood cancer on education, however, such effect modification has rarely been studied [10, 12, 14].

In this comprehensive population-based register study, we sought to examine the educational attainment of childhood cancer survivors in Denmark, Finland, and Sweden, in comparison with that of matched individuals from the general population and of survivors’ siblings, focusing on attainment of upper secondary education in young adulthood. We also aimed to identify vulnerable groups of survivors and to assess educational delay.

## Material and methods

### Design, study population, and data sources

This study is part of the SALiCCS (Socioeconomic Consequences in Adult Life after Childhood Cancer in Scandinavia) research programme, details of which have been published elsewhere [15]. We used a register-based matched cohort design and linked individual information for our study population across various nationwide registries with the unique personal identification number assigned to the residents of all Nordic countries. Denmark, Finland, and Sweden have nationwide registers covering health and social characteristics of the population. The three countries also have similar health care systems and to a large extent common treatment protocols within paediatric oncology, which makes it reasonable to combine data from these countries [15, 16].

We focused on long-term survivors of childhood cancer. All survivors of a first childhood cancer (including non-malignant CNS tumours) in Denmark, Finland, and Sweden born in 1960–1990 (1960–1989 in Finland), diagnosed at ages 0–14 during 1971–2005 (1971–2003 in Finland) who were alive and had not emigrated by the end of the year they turned 25 were eligible (Supplementary Fig. 1). We identified the survivors from the national cancer registers [17] and classified the diagnoses into groups according to the International Classification of Childhood Cancer [18]; we grouped acute lymphoid leukaemia (ALL, defined as group Ia), other leukaemias (Ib-Ie), lymphomas (II), CNS tumours (III), and non-CNS solid tumours (IV-XI) separately.

For each survivor, five individuals, referred to as population comparisons, were randomly sampled from the national population registries and individually matched by sex, year of birth and country of residence (region in Sweden). All biological and adopted siblings with an age difference of ≤5 years from the corresponding survivor were identified as a second comparison group to account for unmeasured genetic and familial background. Individuals in both comparison groups had to be cancer-free up to the age of 20 years (Supplementary Fig. 1).

As a cancer predisposition syndrome may confound associations with educational outcomes, we excluded individuals with Down syndrome, neurofibromatosis, or tuberous sclerosis. For survivors and population comparisons, the reference date was defined as the date of cancer diagnosis of the survivor. For siblings, the reference date was defined as the date on which the sibling was of the same age as the corresponding survivor at cancer diagnosis.

### Outcome assessment

Annual individual information on highest attained educational level was retrieved from national registers administrated by statistical institutes [19–21] for the period 1985–2015 (1985, 1987–2014 in Finland). Educational level was categorised according to the International Standard Classification of Education (ISCED) as no education registered, lower secondary education or less (ISCED ≤ 2), upper secondary and non-tertiary post-secondary education (ISCED 3–4) and tertiary education (ISCED ≥ 5) [22]. In Finland, education levels below ISCED 3 are not registered; as virtually all Finnish children attend comprehensive school, missing information from Finland was considered to be lower secondary education or less.

We defined our main outcome as attainment of upper secondary education (ISCED 3) by age 25. We also assessed attainment of upper secondary education without delay, defined as having attained ISCED 3 or higher by age 19 in Finland and Sweden, and by age 20 in Denmark. Although the education systems in the three countries are overall similar, the difference in age reflects some diversity and different traditions [23].

### Covariates

Age at the reference date was categorised similarly to Nordic school ages as preschool and younger (ages 0–6), lower stage of comprehensive school (ages 7–11), and higher stage of comprehensive school (ages 12–14). The highest attained parental educational level was considered that obtained by biological parents the year before the reference year and grouped as attainment of upper secondary education (ISCED 3) or not.

We collected information from the national patient registers on in- and outpatient hospital care. Time spent in hospital during and after diagnosis (defined as inpatient care within 5 years after the reference date) was used as an indicator of length of treatment and occurrence of complications. The variable was dichotomized (short, long) by the median value in each group of cancer diagnoses, country, and calendar period. We also assessed whether the individual had any hospital contacts for specified somatic disorders (Supplementary Table 1) at ages 20–24 years, categorised as none, cancer-related (i.e., main diagnosis is cancer), and other diagnoses. These categories have been used in previous studies of childhood cancer survivors as conditions related to somatic late effects [3]. Additionally, we assessed hospital contacts for psychiatric diagnoses at ages 20–24 (none, any).

### Statistical analysis

We fitted logistic regression models to estimate odds ratios (ORs) and 95% confidence intervals (CIs) for the risk of not having attained upper secondary education by age 25. For the comparison of survivors with population comparisons, we used unconditional logistic regression models, crude and adjusted for the matching factors (country, sex, age (0–6, 7–11, 12–15) and reference year (10-year intervals)). Survivors and siblings were compared in conditional logistic regression models, crude and adjusted for sex and reference year, to enable comparisons within each sibling set. All the main analyses were also conducted separately for ALL, other leukaemias, lymphomas, CNS tumours, and non-CNS solid tumours. Stratified analyses were conducted by sex, age (0–6, 7–11, 12–15), reference period (1971–1989, 1990–2005), country, time spent in hospital (short, long), and somatic and psychiatric hospital contacts, performed separately for all cancers combined, ALL, CNS, and non-CNS solid tumours. As an additional analysis, we simultaneously stratified by age and reference year among children with ALL.

In comparing the educational attainment of survivors and population comparisons, we assessed the role of parental education by adjusting for it as a potential confounder and conducted stratified analyses. We also assessed potential interaction on the additive scale between cancer survivorship and parental education on the effect of educational attainment, by calculating the relative excess risk due to the interaction (RERI) with 95%CI [24]. RERI is an estimate of the joint effect of survivorship and having parents with a low level of education (hereinafter “low education”), i.e., the effect that is additional to the sum of the two individual factors on educational attainment.

Among individuals who had attained upper secondary education by age 25, we compared the probability of attainment without delay in logistic regression analysis, modelled in the same way as for the main analyses.

Analyses were conducted with SAS 9.4 and Stata 14. The level of statistical significance was set to <0.05.

## Results

Our study population comprised 7629 survivors (4085 males and 3544 females), 35,411 population comparisons and 6114 siblings (Table 1 and Supplementary Fig. 1). Of the survivors, 47.5% were diagnosed with cancer before school age (0–6 years, Table 1).

**Table 1:** Characteristics of childhood cancer survivors diagnosed at ages 0–14 and alive at age 25, their population comparisons and siblings.

	Survivors: All diagnoses combined^a^		Survivors: ALL		Survivors: Other leukaemia		Survivors: Lymphoma		Survivors: CNS tumours		Survivors: Non-CNS solid tumours		Population comparisons		Siblings	
	*n*	%	*n*	%	*n*	%	*n*	%	*n*	%	*n*	%	*n*	%	*n*	%
Total	7629	100	1793	100	218	100	993	100	2067	100	2396	100	35411	100	6114	100
Country of residence																
Denmark	1930	25.3	459	25.6	55	25.2	233	23.5	539	26.1	625	26.1	8297	23.4	1380	22.6
Finland	2032	26.6	520	29.0	63	28.9	270	27.2	434	21.0	727	30.3	9878	27.9	1658	27.1
Sweden	3667	48.1	814	45.4	100	45.9	490	49.3	1094	52.9	1044	43.6	17236	48.7	3076	50.3
Sex																
Males	4085	53.5	938	52.3	96	44.0	652	65.7	1124	54.4	1191	49.7	19049	53.8	3141	51.4
Females	3544	46.5	855	47.7	122	56.0	341	34.3	943	45.6	1205	50.3	16362	46.2	2973	48.6
Year of birth																
1960–1969	1050	13.8	124	6.9	35	16.1	146	14.7	356	17.2	354	14.8	4956	14.0	1016	16.6
1970–1979	2765	36.2	639	35.6	66	30.3	349	35.1	709	34.3	928	38.7	12806	36.2	2225	36.4
1980–1990	3814	50.0	1030	57.4	117	53.7	498	50.2	1002	48.5	1114	46.5	17649	49.8	2873	47.0
Age at diagnosis/reference date (years)																
0–6	3620	47.5	1178	65.7	85	39.0	259	26.1	812	39.3	1197	50.0	16466	46.5	2794	45.7
7–11	2088	27.4	395	22.0	58	26.6	347	34.9	708	34.3	549	22.9	9856	27.8	1733	28.3
12–15	1921	25.2	220	12.3	75	34.4	387	39.0	547	26.5	650	27.1	9089	25.7	1587	26.0
Calendar period of diagnosis/reference date																
1971–1979	1789	23.4	341	19.0	45	20.6	165	16.6	489	23.7	691	28.8	8225	23.2	1432	23.4
1980–1989	3049	40.0	816	45.5	70	32.1	375	37.8	772	37.3	942	39.3	14147	40.0	2653	43.4
1990–1999	2400	31.5	575	32.1	86	39.4	373	37.6	706	34.2	633	26.4	11202	31.6	1801	29.5
2000–2005	391	5.1	61	3.4	17	7.8	80	8.1	100	4.8	130	5.4	1837	5.2	228	3.7
Highest parental education level^b^																
Lower secondary or less	1501	21.1	332	19.8	34	16.8	215	22.7	394	20.1	483	22.2	7219	21.9	1293	22.7
Upper secondary or higher	5060	71.0	1208	71.9	154	76.2	671	70.9	1414	72.0	1518	69.7	23050	69.8	3898	68.3
Time spent in hospital during and after diagnosis^c^																
Median	10.6		20.1		27.1		11.4		5.6		6.5		0		0	
Somatic hospital contacts in age 20–24^d^																
None	3536	46.3	860	48.0	72	33.0	436	43.9	837	40.5	1242	51.8	24575	69.4	4213	68.9
Any																
Cancer-related	1966	25.8	409	22.8	85	39.0	325	32.7	606	29.3	509	21.2	575	1.6	134	2.2
Other	2127	27.9	524	29.2	61	28.0	232	23.4	624	30.2	645	26.9	10261	29.0	1767	28.9
Psychiatric hospital contacts in age 20–24^e^																
None	7067	92.6	1664	92.8	201	92.2	925	93.2	1896	91.7	2228	93.0	33443	94.4	5753	94.1
Any	562	7.4	129	7.2	17	7.8	68	6.8	171	8.3	168	7.0	1968	5.6	361	5.9

*ALL* Acute lymphoid leukaemia, *CNS* Central nervous system.

^a^Other and non-specified tumours were not included in cancer type specific analyses, therefore, the cancer type specific numbers do not add up to the total.

^b^Numbers do not add up because of missing values. For Finland, valid information is restricted to individuals with reference year 1981 and later.

^c^Average number of days in hospital per year during the first 5 years after reference date.

^d^Based on main diagnosis, see Supplementary Table 1 for included ICD-codes.

^e^Based on main diagnosis ICD-8: 290–315; ICD-9: 290–319; ICD-10: F00–F99.

**Fig. 1:**
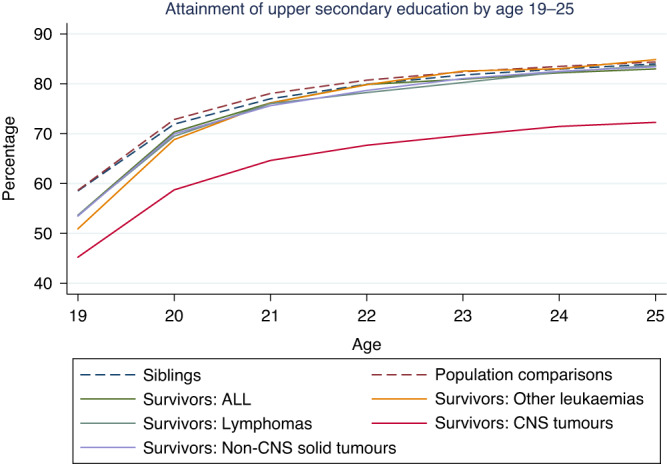
Attainment of upper secondary education. Proportion of survivors, population comparisons and siblings having attained upper secondary education by the respective ages 19–25, stratified by cancer type.

In total, 6127 (80.3%) survivors, 29,880 (84.4%) population comparisons, and 5135 (84.0%) siblings had attained upper secondary education by age 25 (Table 2, Fig. 1). The overall adjusted ORs of the risk of not having attained upper secondary education by age 25, comparing survivors with population comparisons and siblings were 1.32 (95% CI: 1.23–1.40) and 1.57 (95% CI: 1.40–1.77), respectively. The associations differed substantially by diagnostic group and were strongest among survivors of CNS tumours (OR_Surv vs PopComp_ 2.05 (95% CI: 1.83–2.29); OR_Surv vs Sib_ 2.72 (95% CI: 2.19–3.39)) and less pronounced among survivors of ALL (OR_Surv vs PopComp_ 1.15 (95% CI: 1.00–1.33); OR_Surv vs Sib_ 1.27 (95% CI: 0.98–1.65)), while no associations were apparent for survivors of other leukaemias, lymphomas, or non-CNS solid tumours when compared with population comparisons (Table 2). In general, the point estimates were somewhat more elevated when survivors were compared with their siblings but also less precise. As the estimates from the crude and adjusted analyses were similar, only adjusted estimates are shown.

**Table 2:** Attainment of upper secondary education among childhood cancer survivors, population comparisons and siblings: proportions, odds ratios and 95% confidence intervals.

	All diagnoses combined	ALL	Other leukaemia	Lymphoma	CNS tumours	Non-CNS solid tumours	Population comparisons	Siblings
Attainment of upper secondary education by age 25, *n* (%)								
Yes	6127 (80.3)	1488 (83.0)	185 (84.9)	831 (83.7)	1494 (72.3)	1999 (83.4)	29880 (84.4)	5135 (84.0)
No	1502 (19.7)	305 (17.0)	33 (15.1)	162 (16.3)	573 (27.7)	397 (16.6)	5531 (15.6)	979 (16.0)
Likelihood of not having attained upper secondary education by age 25, adjusted OR (95% CI)								
Survivors vs population comparisons^a^	1.32 (1.23–1.40)	1.15 (1.00–1.33)	1.02 (0.67–1.55)	1.01 (0.84–1.22)	2.05 (1.83–2.29)	1.03 (0.91–1.16)	NA	NA
Survivors vs siblings^b^	1.57 (1.40–1.77)	1.27 (0.98–1.65)	1.18 (0.56–2.47)	1.07 (0.76–1.50)	2.72 (2.19–3.39)	1.17 (0.94–1.47)	NA	NA
Time of attainment (among individuals that have attained an upper secondary education by age 25)^c^, *n* (%)								
Without delay^d^	4361 (71.3)	1083 (72.8)	123 (66.5)	589 (71.0)	1011 (67.7)	1451 (72.8)	18801 (77.2)	3326 (77.7)
1 year delay	978 (16.0)	226 (15.2)	37 (20.0)	140 (16.9)	265 (17.7)	294 (14.7)	3198 (13.1)	548 (12.8)
2 years delay	340 (5.6)	96 (6.5)	10 (5.4)	36 (4.3)	92 (6.2)	103 (5.2)	1028 (4.2)	177 (4.1)
3–6 years delay	441 (7.2)	82 (5.5)	15 (8.1)	65 (7.8)	126 (8.4)	146 (7.3)	1327 (5.4)	229 (5.4)
Likelihood of having attained upper secondary education without delay, adjusted OR (95% CI)								
Survivors vs population comparisons^a^	0.75 (0.70–0.80)	0.79 (0.69–0.90)	0.70 (0.49–1.00)	0.70 (0.59–0.83)	0.61 (0.54–0.69)	0.87 (0.77–0.97)	NA	NA
Survivors vs siblings^b^	0.72 (0.64–0.81)	0.76 (0.60–0.97)	0.55 (0.29–1.04)	0.76 (0.54–1.08)	0.53 (0.42–0.68)	0.87 (0.70–1.07)	NA	NA

*ALL* Acute lymphoid leukaemia, *CI* Confidence Interval, *CNS* Central nervous system, *NA* Not applicable, *OR* Odds Ratio

^a^Unmatched analyses, adjusted for country, sex, age, calendar period of diagnosis.

^b^Matched analyses, adjusted for sex and calendar period of diagnosis.

^c^Only survivors with at least one population comparison or sibling that has achieved upper secondary education by age 25 are included. Population comparisons and siblings are only included if there is a survivor in the set.

^d^In Sweden and Finland at age 19, in Denmark at age 20.

The OR of not having attained upper secondary education by age 25, comparing survivors with population comparisons, differed by sex, age, reference year, time spent in hospital, and hospital contacts (Table 3 and Supplementary Table 2). After stratification by age at diagnosis, the association for survivors of CNS tumours was strongest among those diagnosed before school age, while for survivors of ALL the most pronounced association was seen in the age group 12–14 (Table 3). The lower educational attainment of survivors of ALL than of population comparisons was confined to survivors of cancers diagnosed in 1971–1989, except for ALL diagnosed after age 12: in this age group, lower education attainment among survivors was observed in both periods (Supplementary Table 3). Stronger associations were found for survivors who had spent more time in hospital during and after diagnosis (OR_Surv vs PopComp_ 1.61, 95% CI: 1.48–1.76), or had hospital contacts in early adulthood; this pattern was consistent across diagnostic groups and was particularly pronounced among survivors who had psychiatric hospital contacts in young adulthood (OR_Surv vs PopComp_ 4.00, 95% CI: 3.26–4.90, Table 3). Survivors of leukaemia (Table 3) and non-CNS solid tumours (Supplementary Table 2) who had spent less time in hospital during and after diagnosis or had no hospital contacts for specified conditions in early adulthood had similar odds of attaining upper secondary education at age 25 as population comparisons.

**Table 3:** Likelihood of not having attained upper secondary education by age 25 among childhood cancer survivors and population comparisons, stratified by potential effect modifiers and mediators; odds ratios and 95% confidence intervals.

	All diagnoses combined			ALL			CNS-tumours		
	Survivors n_Have not attained_ (%)	Population comparisons n_Have not attained_ (%)	Adjusted OR (95% CI)^a^	Survivors n_Have not attained_ (%)	Population comparisons n_Have not attained_ (%)	Adjusted OR (95% CI)^a^	Survivors n_Have not attained_ (%)	Population comparisons n_Have not attained_ (%)	Adjusted OR (95% CI)^a^
Country									
Denmark	640 (33.2)	2103 (25.3)	1.47 (1.32–1.63)	130 (28.3)	510 (26.1)	1.12 (0.89–1.41)	264 (49.0)	608 (26.0)	2.77 (2.28–3.36)
Finland	334 (16.4)	1348 (13.6)	1.25 (1.09–1.42)	74 (14.2)	318 (12.6)	1.15 (0.88–1.52)	95 (21.9)	300 (14.2)	1.70 (1.31–2.20)
Sweden	528 (14.4)	2080 (12.1)	1.23 (1.11–1.37)	101 (12.4)	404 (10.7)	1.19 (0.94–1.50)	214 (19.6)	629 (12.2)	1.77 (1.49–2.10)
Sex									
Males	838 (20.5)	3343 (17.5)	1.20 (1.10–1.31)	165 (17.6)	741 (17.1)	1.02 (0.84–1.23)	323 (28.7)	939 (17.8)	1.88 (1.62–2.19)
Females	664 (18.7)	2188 (13.4)	1.48 (1.34–1.63)	140 (16.4)	491 (12.5)	1.35 (1.10–1.67)	250 (26.5)	598 (13.8)	2.30 (1.93–2.73)
Age at diagnosis/reference date									
0–6	726 (20.1)	2364 (14.3)	1.49 (1.36–1.64)	194 (16.5)	789 (14.6)	1.13 (0.95–1.35)	271 (33.4)	511 (13.7)	3.27 (2.73–3.91)
7–11	430 (20.6)	1568 (16.1)	1.35 (1.19–1.52)	62 (15.7)	288 (15.7)	0.98 (0.73–1.33)	189 (26.7)	566 (17.1)	1.78 (1.47–2.17)
12–14	346 (18.0)	1599 (17.6)	1.02 (0.90–1.16)	49 (22.3)	155 (15.0)	1.61 (1.11–2.33)	113 (20.7)	460 (17.8)	1.20 (0.95–1.52)
Calendar period of diagnosis/reference date									
1971–1989	1010 (20.9)	3618 (16.2)	1.36 (1.26–1.47)	208 (18.0)	805 (15.1)	1.21 (1.02–1.44)	392 (31.1)	980 (16.8)	2.27 (1.98–2.62)
1990–2005	492 (17.6)	1913 (14.7)	1.23 (1.11–1.38)	97 (15.3)	427 (14.6)	1.04 (0.82–1.33)	181 (22.5)	557 (14.8)	1.68 (1.39–2.04)
Highest parental education level^b^									
Lower secondary or less	408 (27.2)	1757 (26.3)	1.10 (0.97–1.26)	75 (22.6)	370 (26.0)	0.96 (0.71–1.29)	133 (33.8)	488 (25.8)	1.63 (1.28–2.08)
Upper secondary or higher	860 (17.0)	2750 (12.4)	1.44 (1.32–1.57)	190 (15.7)	620 (11.8)	1.36 (1.14–1.63)	347 (24.5)	776 (12.7)	2.25 (1.94–2.60)
Time spent in hospital during and after diagnosis^c, d^									
Short	602 (16.4)	2664 (15.6)	1.05 (0.95–1.15)	124 (14.2)	605 (15.0)	0.92 (0.75–1.14)	205 (20.8)	761 (16.5)	1.33 (1.11–1.58)
Long	861 (23.0)	2715 (15.6)	1.61 (1.48–1.76)	175 (19.6)	607 (14.8)	1.39 (1.15–1.68)	352 (34.5)	733 (15.6)	2.98 (2.55–3.48)
Somatic hospital contacts in age 20–24^d, e^									
None	601 (17.0)	2611 (15.9)	1.07 (0.97–1.18)	136 (15.8)	591 (14.9)	1.05 (0.86–1.29)	178 (21.3)	638 (16.3)	1.38 (1.14–1.68)
Any									
Cancer-related	378 (19.2)	1391 (15.1)	1.33 (1.17–1.51)	68 (16.6)	264 (13.9)	1.23 (0.91–1.65)	175 (28.9)	429 (15.1)	2.36 (1.91–2.91)
Other	523 (24.6)	1529 (15.6)	1.77 (1.58–1.99)	101 (19.3)	377 (15.6)	1.27 (0.99–1.63)	220 (35.3)	470 (16.4)	2.85 (2.35–3.47)
Psychiatric hospital contacts in age 20–24^d, f^									
None	1267 (17.9)	5123 (15.6)	1.17 (1.09–1.25)	254 (15.3)	1144 (14.9)	1.01 (0.87–1.17)	496 (26.2)	1398 (15.8)	1.91 (1.69–2.15)
Any	235 (41.8)	408 (15.8)	4.00 (3.26–4.90)	51 (39.5)	88 (14.9)	3.95 (2.56–6.11)	77 (45.0)	139 (17.6)	4.11 (2.85–5.94)

Analyses are stratified and each strata has its own matched comparison group.

n _Have not attained_ (%) refers to the number and proportion of the population that have not attained upper secondary education by age 25.

*ALL* Acute lymphoid leukaemia, *CI* Confidence Interval, *CNS* Central nervous system, *OR* Odds Ratio.

^a^Unmatched analyses, adjusted for country, sex, age, calendar period of diagnosis.

^b^Restricted to participants with valid information on parental education (for Finland, this information is available only for individuals with reference year 1981 or later).

^c^Based on average number of days in hospital per year during the first 5 years after reference date. Dichotomized by the median value in each group of cancer diagnoses (ALL, Other leukaemia, Lymphoma, CNS tumours, Non-CNS solid tumours), country (Denmark, Finland, Sweden), and calendar period of diagnosis (1971–1979, 1980–1989, 1990–2005).

^d^Stratified on characteristics of the survivors only.

^e^Based on main diagnosis, see Supplementary Table 1 for included ICD-codes.

^f^Based on main diagnosis ICD-8: 290–315; ICD-9: 290–319; ICD-10: F00– F99.

Both survivors and population comparisons with parents who had achieved high education were more likely to attain upper secondary education; however, the difference between survivors and population comparisons was more pronounced for individuals with parents who had a higher education level than among those with parents who had low education (Table 3). Among individuals whose parents had low education, survivors of leukaemia and non-CNS tumours completed upper secondary education to at least the same extent as population comparisons (Table 4). There was no statistically significant additive interaction. Adjustment for parental education as a potential confounder did not appreciably change the effect estimates from those of the main analysis (Supplementary Table 4).

**Table 4:** Interaction analysis between being a cancer survivor and parental education: Likelihood of not having attained upper secondary education by age 25 among childhood cancer survivors and population comparisons; odds ratios and 95% confidence intervals^a^.

	All diagnoses combined		ALL		CNS-tumours		Non-CNS solid tumours	
	*n*	Adj OR (95% CI)^b^	*n*	Adj OR (95% CI)^b^	*n*	Adj OR (95% CI)^b^	*n*	Adj OR (95% CI)^b^
Population comparisons with parents having higher education	22200	1 (ref)	5234	1 (ref)	6111	1 (ref)	6672	1 (ref)
Population comparisons with parents having lower education	6674	2.66 (2.47–2.85)	1425	2.79 (2.40–3.25)	1895	2.44 (2.13–2.79)	2119	2.74 (2.42–3.11)
Survivors with parents having higher education	5060	1.44 (1.33–1.57)	1208	1.36 (1.14–1.63)	1414	2.25 (1.95–2.61)	1518	1.02 (0.86–1.21)
Survivors with parents having lower education	1501	2.90 (2.56–3.29)	332	2.61 (1.97–3.45)	394	3.88 (3.08–4.89)	483	2.83 (2.27–3.52)
RERI^c^	−0.20 (−0.58–0.19)		−0.55 (−1.36–0.26)		0.19 (−0.73–1.11)		0.07 (−0.59–0.73)	

*ALL* Acute lymphoid leukaemia, *CI* Confidence Interval, *CNS* Central nervous system, *OR* Odds Ratio, *RERI* Relative Excess Risk due to Interaction.

^a^Restricted to participants with valid information on parental education (for Finland, this information is available only for individuals with reference year 1981 or later).

^b^Unmatched analyses, adjusted for country, sex, age, calendar period of diagnosis.

^c^RERI = OR_Survivors with parents having lower education_ - OR_Survivors with parents having higher education_ - OR_Population comparisons with parents having lower education_ - 1.

Among individuals who had attained upper secondary education by age 25, survivors were less likely to have completed this without delay; completion of upper secondary education without delay was achieved by 4361 (71.3%) survivors, 18,801 (77.2%) population comparisons and 3326 (77.7%) siblings. The overall adjusted ORs comparing the probability of completing upper secondary education without delay were 0.75 (95% CI: 0.70–0.80) and 0.72 (95% CI: 0.64–0.81) when survivors were compared to population comparisons and siblings, respectively. This pattern was seen in all diagnostic groups (Table 2 and Fig. 1) and in all three countries (Supplementary Table 5 and Supplementary Fig. 2).

Table 5 shows the distribution of educational level among individuals for whom follow-up information was available until age 30. A smaller proportion of the survivors had attained tertiary education by age 30, but the difference was smaller when restricting to individuals who had attained upper secondary education by age 25. For survivors of non-CNS solid tumours, similar proportions of tertiary education were observed compared with population comparisons and siblings (Supplementary Table 6).

**Table 5:** Highest attained education level by age 19, 25 and 30 among survivors diagnosed at ages 0–14 and the two comparison groups^a, b^.

	Age 19			Age 25			Age 30		
	Survivors	Population comparisons	Siblings	Survivors	Population comparisons	Siblings	Survivors	Population comparisons	Siblings
	*n* (%)	*n* (%)	*n* (%)	*n* (%)	*n* (%)	*n* (%)	*n* (%)	*n* (%)	*n* (%)
Total									
Education level									
Lower secondary or less	2707 (49.1)	10365 (41.6)	1757 (42.6)	1109 (20.1)	4068 (16.3)	681 (16.5)	929 (16.9)	3265 (13.1)	539 (13.1)
Upper secondary	2806 (50.9)	14536 (58.4)	2368 (57.4)	3371 (61.1)	15784 (63.4)	2610 (63.3)	2837 (51.5)	12829 (51.5)	2143 (52.0)
Tertiary	NA	NA	NA	1033 (18.7)	5049 (20.3)	834 (20.2)	1747 (31.7)	8807 (35.4)	1443 (35.0)
Restricted to individuals having attained at least upper secondary education at age 25^b^									
Education level									
Upper secondary	NA	NA	NA	3363 (76.5)	12789 (75.5)	2113 (74.2)	2678 (60.9)	9869 (58.3)	1608 (56.5)
Tertiary	NA	NA	NA	1032 (23.5)	4148 (24.5)	733 (25.8)	1717 (39.1)	7068 (41.7)	1238 (43.5)

*NA* Not applicable.

^a^Restricted to individuals that could be followed until age 30, i.e., individuals born 1960–1985 (Finland 1960–1984) and diagnosed 1971–2000 (Finland 1971–1999).

^b^Only survivors with at least one population comparison or sibling that has achieved upper secondary education by age 25 are included. Population comparisons and siblings are only included if there is a survivor in the set.

## Discussion

In this large register-based cohort study nested in the entire populations of three Nordic countries, we observed that, overall, a smaller proportion of childhood cancer survivors had attained upper secondary education by age 25 than population comparisons and siblings. The differences were, however, largely confined to survivors of CNS tumours, while smaller differences were seen for survivors of ALL diagnosed in 1971–1989 or at ages 12–14 years. Survivors of all cancer types experienced delays in education, but survivors of leukaemias other than ALL, lymphomas, and non-CNS solid tumours had caught up with their peers with regard to upper secondary education by age 25. Survivors who had spent more time in hospital during and after their cancer diagnosis or had hospital contacts in early adulthood were at particular risk of not attaining upper secondary education by age 25, especially survivors who had psychiatric hospital contacts. Parental education had a considerable impact on educational attainment in general, however, stratification by parental education showed the largest differences between survivors and population comparisons among those with parents with high education.

The pronounced educational disadvantage of survivors of CNS tumours is well recognised in the literature [4–7], seen even at younger ages [12, 13]. Our study suggests that this group of survivors does not catch up over time as do survivors of other types of cancer, in accordance with previous findings in Switzerland [9]. We found that survivors of CNS tumours diagnosed before school age were particularly vulnerable, with a somewhat stronger association among females and individuals diagnosed in the early calendar period, similar to previous studies [12, 13, 25]. However, survivors of CNS tumours were less likely to attain upper secondary education by age 25 than their peers in all the strata investigated, highlighting the need to support this group.

Survivors of ALL diagnosed in 1971–1989 also had a lower level of education than their peers at the age of 25. This is probably due to the use of cranial radiotherapy during that period [26]; previous studies also found lower educational attainment among survivors of leukaemia who were treated with irradiation [10, 27–29]. The difference between calendar periods was seen mainly among younger children, who are more sensitive to irradiation [30]. We also found that survivors of ALL diagnosed at ages 12–14 were at risk of poorer educational outcomes in both calendar periods. This finding is somewhat unexpected, although some other studies suggested that also older children with leukaemia are at risk of lower educational attainment [9, 10].

We found that survivors who had spent more time in hospital during and after diagnosis and survivors with more hospital contacts in early adulthood, i.e., after the original cancer treatment, were at higher risk of not attaining upper secondary education by age 25. This is in line with previous research of more specific health problems. For example, in the British Childhood Cancer Survivor Study, epilepsy influenced education negatively [27]. A German study of survivors of adolescent cancer suggested that visual or hearing late effects as well as neuropsychological late effects were risk factors for poorer educational attainment at different levels, while increasing length of treatment was borderline significant [31]. This result may also reflect that survivors experiencing a relapse or a second primary malignancy, who therefore have more contacts with hospitals, is a vulnerable group with regard to educational achievements. However, such specific conclusions require further investigation. In our study, the risk of poorer educational outcomes was particularly pronounced among survivors with psychiatric hospital contacts; this finding is important with regard to potential targeted interventions, although this is a small group of survivors as seen in the current study as well as in previous research from our group [11].

It has been shown previously that survivors with parents who had low education had worse educational outcomes than survivors with parents with high education [9, 12, 13], which reflects the pattern in the general population and is also seen in our study population. In addition to existing literature, we observed that the difference between survivors and comparisons was largest among children of parents with high education. This finding runs contrary to sociological theories of “compensatory advantage”, which argue that highly educated parents have more resources to counteract negative childhood circumstances [14], and suggests that childhood cancer is more disruptive for the educational trajectories of children of highly educated parents. Also, as it is less common that children with higher educated parents do not complete upper secondary education, the relative and absolute differences in this group become more pronounced. Indeed, survivors of ALL and non-CNS solid tumours whose parents had low education completed upper secondary education to the same extent or even more frequently than the corresponding population comparisons, which suggests that these groups of survivors gained from provided support. For survivors of CNS tumours, however, the combination of experiencing a cancer diagnosis as a child and having parents with lower education was associated with a particularly high risk of not completing upper secondary education before age 25.

Survivors of non-CNS solid tumours who had attained an upper secondary education attained tertiary education to a similar extent as their peers. A previous meta-analysis showed that survivors without CNS involvement had no disadvantage in achieving tertiary education but highlighted the risk of bias from non-participation [7]. This bias was not a concern in the present study, which strengthens the conclusion that, for this group, childhood cancer can disrupt education primarily in the early stages but has no further impact on later educational transitions. However, for other groups of survivors there seem to be a difference in achievement of tertiary education.

Our study is unique in that it combined high-quality register data from three Nordic countries and included comparisons with both the general population and siblings. Use of siblings as a second reference group controlled for confounding from shared familial and social backgrounds and strengthens the validity of our findings, although these analyses had less statistical power, as only data of siblings discordant for the outcome contributed to the estimates. The large population allowed subgroup analyses, which is important for identifying survivors who would benefit most from targeted support. However, although the whole population of survivors in Denmark, Finland, and Sweden were included, statistical power limited analyses of, for example, more defined cancer types (e.g., specific types of non-CNS solid tumours). As information on educational attainment was obtained from national population-based registers, there was no risk of bias due to self-reporting, non-participation, or selection. Further, the longitudinal information was obtained in the same way for the survivor and comparison groups.

The register-based design and the three-country wide inclusion have many advantages but also some limitations. We had no information on the reasons for delayed graduation, which are not necessarily related to educational problems. We also lacked information on treatment, especially cranial radiation therapy, an established risk factor for poorer educational achievement [4]. Inclusion in future studies of more clinical information would improve understanding of the underlying mechanisms and help to identify vulnerable survivors. Our study population came from three Nordic countries which have remarkably comparable, although not identical, health-, social- and educational systems. The matched design took differences between countries into consideration. Country specific analyses showed similar results, although effect estimates differed somewhat in magnitude. This could reflect true differences across countries but may also be a result of random variation. It is important to acknowledge differences between countries when interpreting the findings, but also the overall Nordic context. Support in school as well as the overall social- and educational systems will impact survivors’ opportunities after a cancer diagnosis, and it is therefore challenging to directly generalise our findings to other countries. We included survivors diagnosed over a long period of time during which treatment regimens have changed, and our results may not be applicable to children undergoing cancer treatment at present. Our findings highlight the importance of continued follow-up of late effects and socioeconomic consequences also among more recently treated survivors.

In this three-country wide register-based cohort study, we demonstrate that, although survivors of childhood cancer are more likely than their peers to experience delays in upper secondary education, many had caught up by the age of 25. Except for survivors of CNS tumours, survivors attained upper secondary education to almost the same extent as their peers. Parental education played an important role also in survivors’ educational attainment and modified the associations. In addition to the vulnerable group of survivors of CNS tumours, we identified survivors who had spent more time in hospital during and after diagnosis and survivors with hospital contacts, particularly for psychiatric diseases, in early adulthood as risk groups for educational difficulties. These findings add to the existing literature and recently published guidelines for surveillance [8] and enhance a possibility of identifying survivors who need additional educational support, both close to the diagnosis and at later follow-up visits.

### Supplementary information

Supplementary material

## Data Availability

The data that support the information of this manuscript were accessed remotely on a secure platform at Statistics Denmark. Pseudonymized individual-level data were obtained from national registry holders after ethical approval (where applicable) and secrecy assessment. According to Danish, Finnish and Swedish laws and regulations, individual-level sensitive data can only be made available for researchers who fulfil legal requirements for access to personal sensitive data. Please contact Jeanette Falck Winther (jeanette@cancer.dk), the Principal Investigator of the SALiCCS research programme, for further questions about data access.
