# Supplementary material for: Educational attainment in survivors of childhood cancer in Denmark, Finland, and Sweden
Source: Br J Cancer. 2023 Nov 22;130(2):260–8. doi: 10.1038/s41416-023-02499-1 (PMC10803319; doi:10.1038/s41416-023-02499-1)
Supplement: Supplementary file 1 — Supplementary material [file 41416_2023_2499_MOESM1_ESM.pdf]

Supplementary material

Educational attainment in survivors of childhood cancer in Denmark, Finland, and Sweden

Mogensen H, Tettamanti G, Frederiksen LE, Talbäck M, Härkonen J, Modig K, Pedersen C, Krøyer A, Hirvonen E, Kyrönlahti A, Heyman M, Holmqvist AS, Hasle H, Madanat-Harjuoja L, Malila N, Winther JF, Erdmann F, Feychting M.

Supplementary Table 1. Grouping of somatic diseases.

Supplementary Table 2. Non-CNS solid tumours: Likelihood of not having attained upper secondary education by age 25 among childhood cancer survivors and population comparisons, stratified by potential effect modifiers and mediators; odds ratios and 95% confidence intervals.

Supplementary Table 3. Likelihood of not having attained upper secondary education by age 25 among survivors of ALL and population comparisons, stratified by age and calendar period of diagnosis; odds ratios and 95% confidence intervals.

Supplementary Table 4. Attainment of upper secondary education by age 25 among childhood cancer survivors and population comparisons, odds ratios and 95% confidence intervals additionally adjusted for parental education.

Supplementary Table 5. Attainment of upper secondary education among childhood cancer survivors (all diagnoses combined) and population comparisons; proportions, odds ratios and 95% confidence intervals, stratified by country.

Supplementary Table 6. Highest attained education level by age 19, 25 and 30 among survivors diagnosed at ages 0-14, population comparisons and siblings, by diagnostic group.

Supplementary Figure 1. Flow chart of the inclusion and exclusion criteria of childhood cancer survivors, population comparisons and siblings.

Supplementary Figure 2. Proportion of survivors, population comparisons and siblings having attained upper secondary education by the respective ages 19-25, stratified by country.

Supplementary Table 1. Grouping of somatic diseases[a]

	ICD-8	ICD-9	ICD-10
**Group 1: Infectious and parasitic diseases**			
Intestinal infectious diseases	000-009	001-009	A00-A09
Tuberculosis	010-019	010-018	A15-A19
Sepsis	038	038	A40-A41
Erysipilas	035	035	A46
Other bacterial diseases	030-034, 036-037, 039	030-034, 036-037, 039-041	A30-A39, A42-A45, A47-A49
Enterovirus diseases of CNS	040-046, 062-066	045-049, 062-064	A80-A81, A83-A89
Herpes zoster	053	053	B02
Other viral diseases with exanthem	050-052, 054-057	050-052, 054-057	B00-B01, B03-B09
Infectious hepatitis, HIV and other viral diseases	070-079	042, 070-079	A70-A71, A82, B15-B34
Syphilis and other venereal diseases	090-104	090-099	A50-A70, A74
Mycoses	110-117	110-118	B35-B49
Other infectious and parasitic diseases	020-027, 060-061, 067-068, 080-089, 120-129, 130-136	020-027, 060-061, 065-066, 080-088, 100-104, 120-136	A20-A28, A75-A79, A90-A99, B50-B83, B85-B99
**Group 2: Malignant and benign neoplasms**			
Cancer of buccal cavity and pharynx	140-149	140-149	C00-C14, C46.2
Cancer of digestive organs	150-159	150-159	C15-C26
Cancer of respiratory system and intrathoracic organs	160-163, 197	160-165, 197	C30-C39, C45.0
Cancer of bones, joints and articular cartilage	170	170	C40-C41
Malignant melanoma of skin	172-173	172-173, 176	C43
Mesothelium and connective tissue	171	171	C45.1-C45.9, C46.1, C46.3, C46.7, C46.8, C46.9, C47-C49, B21.0
Cancer of breast	174	174-175	C50
Cancer of female genital organs incl. skin	180-184	179-184	C51-C58
Cancer of male genital organs incl. skin	185-187	185-187	C60-C63
Cancer of urinary tract	188-189	188-189	C64-C68, D30.1-D30.9, D41.4

Cancer of eye, brain and other parts of central nervous system	190-193	190-192	C69-C72, C75.1-C75.3, D32-D33, D35.2-D35.4, D42-D43, D44.3-D44.5
Cancer of endocrine organs	194	193-194	C73-C74, C75.0, C75.4-C75.9
Malignant lymphomas	196, 200-202	196, 200-202	C81, C82-C85, C88.3-C88.9
Multiple myeloma	203	203	C90, C88.0-C88.2
Leukaemia	204-207	204-208	C91-C96
Ill-defined and unspecified cancer	195, 198, 199, 208, 209, 230-239	195, 198, 199	C76-C80, C44
Benign neoplasms	210-222, 223.0, 224, 226, 227-228, 225, 226	210-222, 223.0, 224, 225-229, 233, 235-239	D00-D08, D09.2-D30.0, D31, D34, D35.0-D35.1, D35.5-D35.9, D10-D48
**Group 3: Endocrine disorders, nutritional deficiencies and other metabolic diseases**			
Diseases of the thyroid gland	240-242, 244-246	240-242, 244-246	E01-E02, E03.2-E03.9, E04-E07
Diabetes mellitus	249, 250	250	E10-E14
Other disorders of glucose regulation and pancreatic internal secretion	251	251	E15-E16
Pituitary hypofunction	253.1	253.2-253.3, 253.5	E23.0-E23.3
Ovarian dysfunction	256	256	E28
Testicular dysfunction	257	257	E29
Disorders of other endocrine organs	252, 253.0, 253.2-253.9, 254-255, 258	252, 253.0-253.1, 253.4, 253.6-253.9, 254, 255, 258-259, 271	E20-E22, E23.6-E23.9, E24-E27, E30-E35
Nutritional deficiencies	260-269	260-269	E40-E64
Other metabolic disorders	275.4-275.9, 276, 278-279	273, 275, 276, 277.1-277.9	E73, E86-E90
Male sterility	606	606	N46
Abnormal menstruation	626	626	N91-N92
Female infertility	628	628	N97
Other disorders of female reproductive system	627	627	N93-N96, N98, N99
**Group 4: Diseases of nervous system and sense organs**			
Meningitis	320	320-322	G00-G03

Other inflammatory diseases of CNS	321-324	323-326	G04-G09
Multiple sclerosis and other demyelinating diseases of CNS	340-341	340-341	G35-G37
Parkinson disease and other movement disorders	342	332, 333	G20-G22, G24-G26
Epilepsy	345	345	G40-G41
Migraine and other diseases of brain and spinal cord	346-347, 349	346-349	G13, G43-G44, G46-G47
Senile and presenile dementia		331	G30-G32
Diseases of nerves and peripheral ganglia	344, 350-358	350-359	G50-G59, G61-G73, G81-G89, G90.0, G90.2-G90.9, G91-G99
Inflammatory and other diseases of the eye	360-373, 375-379	360-365, 367-379	H00-H22, H30-H36, H40-H59
Cataract	374	366	H25-H28
Inflammatory diseases of ear	380-384	380-384	H60-H75
Ménière disease and orthosclerosis	385-386	386-387	H80-H82
Other diseases of ear and deafness	387-389	385, 388-389	H83-H95
**Group 5: Diseases of circulatory system**			
Acute rheumatic fever	390-392	390-392	I00-I02
Chronic rheumatic heart disease	393-398	393-398	I05-I09
Hypertensive disease	400-404	401-405	I10-I15
Ischemic heart disease	410-414	410-414, 429.2, 429.7	I20-I25
Pulmonary heart disease	426, 450	415-417	I26-I28
Pericardial, myocardial- and endocardial disease	420-423	420-423, 424.9, 429.0	I30-I33, I38-I41, I51.4
Valvular disease (non-rheumatic)	424	424.0-424.3	I34-I37
Heart failure	425, 427.0-427.1, 428-429	425, 428, 429.1, 429.3, 429.8-429.9	I42-I43, I50, I51.5, I51.7
Conduction disorders	427.2-427.9	426-427	I44-I49
Cerebrovascular disease	430-438	430-438	I60-I69, G45
Diseases of arteries, arterioles and capillaries	440-445, 447-448	440-445, 447-448	I70-I79
Venous and lymphatic disease	451-457	451-457	I80-I89

Other complications of the circulatory system	446, 458	429.4-429.6, 446, 458-459	I51.0-I51.3, I51.6, I51.8-I51.9, I52, I95-I99, M30
**Group 6: Diseases of respiratory system**			
Influenza	470-474	487	J10-J11
Acute upper respiratory infections	460-465	460-465	J00-J06
Other disorders of upper respiratory tract	500-508	470-478	J30-J39
Pneumonia	480-486, 517	480-486	J12-J18
Abscess of lung and pyothorax	510-511, 513	510-511, 513	J85-J86, J90
Bronchitis and emphysema	466, 490-492, 518	466, 490-492, 494, 496, 518.1-518.2	J20-J22, J40-J44, J47, J98.2-J98.3
Asthma	493	493	J45-J46
Lung diseases due to external agents	515-516	495, 500-508	J60-J70
Interstitial pulmonary diseases and pulmonary oedema	514, 519.1	514-516, 518.3-518.4	J81-J84
Pneumothorax	512	512	J93
Respiratory failure	519.0	518.0, 518.5-518.8	J96, J98.1
Other diseases of respiratory system	519.2-519.9	517, 519	J80, J91-J92, J94-J95, J98.0, J98.4-J98.9, J99
**Group 7: Diseases of digestive organs**			
Diseases of the teeth and supporting structures	520-525	520-525	K00-K08
Other diseases of the oral cavity and salivary glands	526-529	526-529	K09-K14
Diseases of oesophagus	530	530	K20-K23
Diseases of stomach and duodenum	531-537	531-537	K25-K31
Appendicitis	540-543	540-543	K35-K38
Hernia of abdominal cavity	550-553	550-553	K40-K46
Noninfective enteritis and colitis	561, 563	555-558	K50-K52
Paralytic ileus and intestinal obstruction	560	560	K56
Diseases of anal and rectal regions	565, 566	565, 566	K60-K62
Diseases of peritoneum	567-568	567, 568	K65-K67

Other diseases of digestive system	562, 564, 569	562, 564, 569, 578-579	K55, K57-K59, K63, K90-K93
Diseases of liver	570-573	570-573	K70-K77
Diseases of gallbladder and biliary ducts	574-576	574-576	K80-K83, K87
Diseases of pancreas	577	577	K85-K86
**Group 8: Diseases of urinary system and genital organs**			
Glomerular diseases	580-583	580-583, 599.7	N00-N01, N03-N05, N02.0-N02.8, N06.0-N06.8, N07.0-N07.8, N08
Acute renal failure	593.1	584	N17, N28.0
Chronic kidney disease	584, 590.0-590.1, 593.0	585-587, 589, 590.0, 590.8, 593.2	N11.8-N11.9, N12, N18-N19, N26-N27, N28.1
Urolithiasis	592, 594	592, 594	N20-N22
Obstructive uropathy	591, 598, 593.3-593.4, 596.2	591, 593.3-593.5, 596.0, 598, 599.6	N11.0-N11.1, N13.0-N13.5, N13.8-N13.9, N32.0, N35
Infections of the urinary system	595, 597, 590.2, 590.9, 599.0	590.1-590.3, 590.9, 595, 597, 599.0	N10, N13.6, N15.1, N16.0, N29.0-N29.1, N30, N33.0, N34, N37.0, N39.0
Other an unspecified disorders of the urinary system	593.2, 593.5, 596.0-596.1, 596.3, 596.9, 599.1-599.2, 599.9	588, 593.0-593.1, 593.6-593.9, 596.1-596.9, 599.1-599.5, 599.81-599.89, 599.9	N02.9, N06.9, N07.9, N13.7, N14, N15.0, N15.8-N15.9, N16.1- N16.8, N23, N25, N28.8-N28.9, N29.8, N31, N32.1-N32.9, N33.8, N36, N37.8, N39.1, N39.2-N39.9
Diseases of prostate	600-602	600-602	N40-N42
Hydrocoele and spermatocoele	603	603	N43
Orchitis and epididymis	604	604	N45
Other diseases of male genital organs	605, 607	605, 607-608	N44, N47-N51
Chronic cystic disease and other diseases of breast	610-611	610-611	N60-N64
Inflammatory diseases of female pelvic organs	612-614, 616.0, 620, 622	614-616	N70-N77
Endometriosis	625.3	617	N80
Noninflammatory disorders of female genital tract	615, 616.1-616.9, 621, 623-624, 625.0-625.2, 625.9, 629	618-625, 629	N81-N90

**Group 9: Diseases of skin and subcutaneous tissue**			
Infections of skin and subcutaneous tissue	680-686	680-686	L00-L08
Other inflammatory conditions of skin and subcutaneous tissue	690-691, 692.0-692.6, 692.8-692.9, 693-698, 708	690-691, 692.00-692.81, 692.83-692.89, 693-698, 702, 708	L10-L57, L59
Radiodermatitis	692.7	692.82	L58
Disorders of skin appendages (hair, nails, sweat glands)	703-706	703-706	L60-L75
Other disorders of the skin and subcutaneous tissue	700-702, 707, 709	700-701, 707, 709	L80-L99
**Group 10: Diseases of bone, joint and soft tissue**			
Arthritis and rheumatism	274, 710-718	274, 710-719, 725-727, 729.0	M00-M19, M79.0
Osteomyelitis and other diseases of bone and joint	720-729	720-724, 730-733	M20-M25, M40-M54, M80-M94
Other diseases of musculoskeletal system	730-738	728, 729.1-729.9, 734-739	M31-M36, M60-M77, M79.1-M79.9, M95-M99

Grouping of somatic diseases based on the ALiCCS research program, published in de Fine Licht et al. 2017. The grouping is previously used in similar studies of childhood cancer survivors (Frederiksen et al. 2022).

[a] We excluded diseases in pregnancy, during birth and perinatal diseases; congenital malformations; certain causes of diseases in the perinatal period and death due to this; symptoms and ill-defined conditions; diseases of blood and blood-forming organs; obesity; external cause of accident; injuries and side-effects of various treatments

Supplementary Table 2. Non-CNS solid tumours: Likelihood of not having attained upper secondary education by age 25 among childhood cancer survivors and population comparisons, stratified by potential effect modifiers and mediators; odds ratios and 95% confidence intervals.

	Non-CNS solid tumours		
	**Survivors**	**Population comparisons**	**Adjusted OR (95% CI)[a]**
	$n_{\text{Have not attained}}$ (%)	$n_{\text{Have not attained}}$ (%)	
**Specific cancer type**			
Sympathetic nervous system tumors	36 (17.5)	149 (16.0)	1.08 (0.71-1.63)
Retinoblastomas	42 (15.6)	165 (13.5)	1.16 (0.80-1.68)
Renal tumours	79 (15.6)	350 (15.1)	1.02 (0.78-1.33)
Hepatic tumours	*	*	0.70 (0.22-2.19)
Malignant bone tumours	44 (16.3)	224 (17.6)	0.90 (0.63-1.29)
Soft tissue sarcomas	64 (17.2)	301 (17.3)	0.98 (0.73-1.32)
Germ cell tumours	42 (18.0)	166 (15.3)	1.21 (0.83-1.77)
Malignant epithelial neoplasms	86 (17.3)	385 (16.5)	1.05 (0.80-1.36)
**Country**			
Denmark	159 (25.4)	669 (25.2)	1.02 (0.83-1.24)
Finland	113 (15.5)	477 (13.5)	1.18 (0.94-1.47)
Sweden	125 (12.0)	620 (12.6)	0.94 (0.77-1.16)
**Sex**			
Males	201 (16.9)	989 (17.9)	0.91 (0.77-1.08)
Females	196 (16.3)	777 (13.9)	1.18 (0.99-1.41)
**Age at diagnosis/reference date (years)**			
0-6	191 (16.0)	806 (14.8)	1.07 (0.90-1.27)
7-11	106 (19.3)	416 (16.3)	1.22 (0.96-1.56)
12-14	100 (15.4)	544 (17.7)	0.84 (0.66-1.06)
**Year of diagnosis/reference date**			
1971-1989	281 (17.2)	1239 (16.5)	1.04 (0.90-1.20)
1990-2005	116 (15.2)	527 (14.8)	1.02 (0.82-1.27)
**Highest parental education level[b]**			
Lower secondary or less	133 (27.5)	582 (27.5)	1.03 (0.82-1.29)
Upper secondary or higher	197 (13.0)	844 (12.6)	1.02 (0.86-1.21)
**Time spent in hospital during and after diagnosis[c]**			
Short	178 (15.5)	834 (15.7)	0.97 (0.81-1.15)
Long	207 (17.8)	867 (16.1)	1.12 (0.94-1.32)
**Somatic hospital contacts in age 20-24[c, d]**			
None	190 (15.3)	940 (16.3)	0.91 (0.77-1.08)
Any			
Cancer-related	80 (15.7)	389 (16.4)	0.93 (0.71-1.22)
Other	127 (19.7)	437 (14.7)	1.40 (1.12-1.75)
**Psychiatric hospital contacts in age 20-24[c, e]**			
None	328 (14.7)	1659 (16.1)	0.88 (0.77-1.01)
Any	69 (41.1)	107 (13.8)	4.51 (3.09-6.58)

Analyses are stratified and each strata has its own matched comparison group.
$n_{\text{Have not attained}}$ (%) refers to the number and proportion of the population that have not attained upper secondary education by age 25.
[a] Unmatched analyses, adjusted for the matching factors (country, sex, age, calendar period of diagnosis).
[b] Restricted to participants with valid information on parental education (for Finland, this information is available only for individuals with reference year 1981 or later).
[c] Stratified on characteristics of the survivors only.
[d] Based on main diagnosis, see Supplementary table 1 for included ICD-codes.
[e] Based on main diagnosis ICD-8: 290-315; ICD-9: 290-319; ICD-10: F00- F99
*Less than five individuals in at least one group.
Abbreviations: CI: Confidence Interval; CNS: Central nervous system; OR: Odds Ratio

Supplementary Table 3. Likelihood of not having attained upper secondary education by age 25 among survivors of ALL and population comparisons, stratified by age and calendar period of diagnosis; odds ratios and 95% confidence intervals.

	Diagnosed age 0-6			Diagnosed age 7-11			Diagnosed age 12-14		
	**Survivors**	**Population comparisons**	**Adjusted OR (95% CI)[a]**	**Survivors**	**Population comparisons**	**Adjusted OR (95% CI)[a]**	**Survivors**	**Population comparisons**	**Adjusted OR (95% CI)[a]**
	$n_{Have\ not\ attained}$ (%)	$n_{Have\ not\ attained}$ (%)		$n_{Have\ not\ attained}$ (%)	$n_{Have\ not\ attained}$ (%)		$n_{Have\ not\ attained}$ (%)	$n_{Have\ not\ attained}$ (%)	
**Calendar period of diagnosis/reference date**									
1971-1989	148 (17.3)	576 (14.7)	1.19 (0.98-1.46)	39 (18.9)	159 (16.6)	1.17 (0.79-1.74)	21 (22.1)	70 (15.9)	1.49 (0.85-2.61)
1990-2005	46 (14.3)	213 (14.5)	0.97 (0.68-1.38)	23 (12.2)	129 (14.8)	0.77 (0.48-1.26)	28 (22.4)	85 (14.4)	1.71 (1.05-2.78)

$n_{Have\ not\ attained}$ (%) refers to the number and proportion of the population that have not attained upper secondary education by age 25.
[a] Unmatched analyses, adjusted for sex and country.
Abbreviations: ALL: Acute lymphoid leukaemia; CI: Confidence Interval; OR: Odds Ratio

Supplementary Table 4. Attainment of upper secondary education by age 25 among childhood cancer survivors and population comparisons, odds ratios and 95% confidence intervals additionally adjusted for parental education.[a]

	**Survivors: All diagnoses combined**		**Survivors: ALL**		**Survivors: CNS tumours**		**Population comparisons**
**Attainment of upper secondary education by age 25, n (%)**							
Yes	5293 (80.7)		1275 (82.8)		1328 (73.5)		24367 (84.4)
No	1268 (19.3)		265 (17.2)		480 (26.5)		4507 (15.6)
**Likelihood of not having attained upper secondary education by age 25, adjusted OR (95% CI)**							
	**Adjusted OR (95% CI)[b]**	**Additionally adjusted OR (95%CI)[c]**	**Adjusted OR (95% CI)[b]**	**Additionally adjusted OR (95%CI)[c]**	**Adjusted OR (95% CI)[b]**	**Additionally adjusted OR (95%CI)[c]**	
Survivors vs population comparisons	1.30 (1.21-1.40)	1.32 (1.23-1.42)	1.20 (1.03-1.39)	1.22 (1.05-1.43)	1.97 (1.74-2.23)	2.05 (1.81-2.32)	

[a] Restricted to participants with valid information on parental education (for Finland, this information is available only for individuals with reference year 1981 or later)
[b] Unmatched analyses, adjusted for country, sex, age, calendar period of diagnosis
[c] Unmatched analyses, adjusted for country, sex, age, calendar period of diagnosis, highest parental education
Abbreviations: ALL: Acute lymphoid leukaemia; CI: Confidence Interval; CNS: Central nervous system; OR: Odds Ratio

Supplementary Table 5. Attainment of upper secondary education among childhood cancer survivors (all diagnoses combined) and population comparisons; proportions, odds ratios and 95% confidence intervals, stratified by country.

	**Denmark**		**Finland**		**Sweden**	
	**Survivors**	**Population comparisons**	**Survivors**	**Population comparisons**	**Survivors**	**Population comparisons**
**Individuals that have attained an upper secondary education by age 25[a]**						
**Time of attainment, n (%)**						
Without delay[b]	784 (61.1)	2650 (63.9)	1123 (66.1)	5249 (73.5)	2454 (78.2)	10902 (83.5)
1 year delay	235 (18.3)	727 (17.5)	318 (18.7)	1045 (14.6)	425 (13.5)	1426 (10.9)
2 years delay	127 (9.9)	376 (9.1)	108 (6.4)	354 (5.0)	105 (3.3)	298 (2.3)
3-6 years delay	138 (10.8)	394 (9.5)	149 (8.8)	496 (6.9)	154 (4.9)	437 (3.3)
**Likelihood of having attained upper secondary education without delay, adjusted OR (95% CI)**						
Survivors vs population comparisons[c]	0.88 (0.78-1.01)		0.70 (0.62-0.78)		0.71 (0.64-0.78)	

[a] Only survivors with at least one population comparison or sibling that has achieved upper secondary education by age 25 are included. Population comparisons and siblings are only included if there is a survivor in the set.
[b] In Sweden and Finland at age 19, in Denmark age 20.
[c] Unmatched analyses, adjusted for country, sex, age, calendar period of diagnosis.
Abbreviations: CI: Confidence Interval; OR: Odds Ratio

Supplementary Table 6. Highest attained education level by age 19, 25 and 30 among survivors diagnosed at ages 0-14, population comparisons and siblings, by diagnostic group[a,b]

	Age 19			Age 25			Age 30		
	**Survivors** n (%)	**Population comparisons** n (%)	**Siblings** n (%)	**Survivors** n (%)	**Population comparisons** n (%)	**Siblings** n (%)	**Survivors** n (%)	**Population comparisons** n (%)	**Siblings** n (%)
ALL									
**Total**									
**Education level**									
Lower secondary or less	600 (48.2)	2255 (40.3)	385 (41.8)	225 (18.1)	867 (15.5)	143 (15.5)	189 (15.2)	708 (12.7)	116 (12.6)
Upper secondary	646 (51.8)	3334 (59.7)	537 (58.2)	777 (62.4)	3452 (61.8)	575 (62.4)	662 (53.1)	2714 (48.6)	460 (49.9)
Tertiary	-	-	-	244 (19.6)	1270 (22.7)	204 (22.1)	395 (31.7)	2167 (38.8)	346 (37.5)
**Restricted to individuals having attained at least upper secondary education at age 25[b]**									
**Education level**									
Upper secondary	-	-	-	776 (76.1)	2870 (73.2)	482 (72.2)	630 (61.8)	2161 (55.1)	367 (54.9)
Tertiary	-	-	-	244 (23.9)	1050 (26.8)	186 (27.8)	390 (38.2)	1759 (44.9)	301 (45.1)
CNS tumours									
**Total**									
**Education level**									
Lower secondary or less	828 (54.5)	2903 (42.4)	512 (43.4)	412 (27.1)	1146 (16.7)	190 (16.1)	361 (23.8)	898 (13.1)	148 (12.5)
Upper secondary	691 (45.5)	3950 (57.6)	669 (56.6)	867 (57.1)	4341 (63.3)	748 (63.3)	784 (51.6)	3614 (52.7)	613 (51.9)
Tertiary	-	-	-	240 (15.8)	1366 (19.9)	243 (20.6)	374 (24.6)	2341 (34.2)	420 (35.6)
**Restricted to individuals having attained at least upper secondary education at age 25[b]**									
**Education level**									
Upper secondary	-	-	-	867 (78.3)	3234 (75.6)	557 (74.0)	737 (66.6)	2539 (59.4)	422 (56.0)
Tertiary	-	-	-	240 (21.7)	1044 (24.4)	196 (26.0)	370 (33.4)	1739 (40.6)	331 (44.0)
Non-CNS solid tumours									
**Total**									
**Education level**									
Lower secondary or less	822 (46.6)	3410 (42.9)	536 (42.5)	306 (17.3)	1328 (16.7)	206 (16.3)	244 (13.8)	1065 (13.4)	161 (12.8)
Upper secondary	942 (53.4)	4537 (57.1)	726 (57.5)	1106 (62.7)	5079 (63.9)	794 (62.9)	880 (49.9)	4106 (51.7)	661 (52.4)
Tertiary	-	-	-	352 (20.0)	1540 (19.4)	262 (20.8)	640 (36.3)	2776 (34.9)	440 (34.9)
**Restricted to individuals having attained at least upper secondary education at age 25[b]**									
**Education level**									
Upper secondary	-	-	-	1100 (75.8)	4231 (76.3)	677 (74.1)	824 (56.8)	3248 (58.5)	520 (56.9)
Tertiary	-	-	-	351 (24.2)	1317 (23.7)	237 (25.9)	627 (43.2)	2300 (41.5)	394 (43.1)

[a] Restricted to individuals that could be followed until age 30, i.e. individuals born 1960-1985 (Finland 1960-1984) and diagnosed 1971-2000 (Finland 1971-1999).
[b] Only survivors with at least one population comparison or sibling that has achieved upper secondary education by age 25 are included. Population comparisons and siblings are only included if there is a survivor in the set.
Abbreviations: ALL: Acute lymphoid leukaemia; CNS: Central nervous system

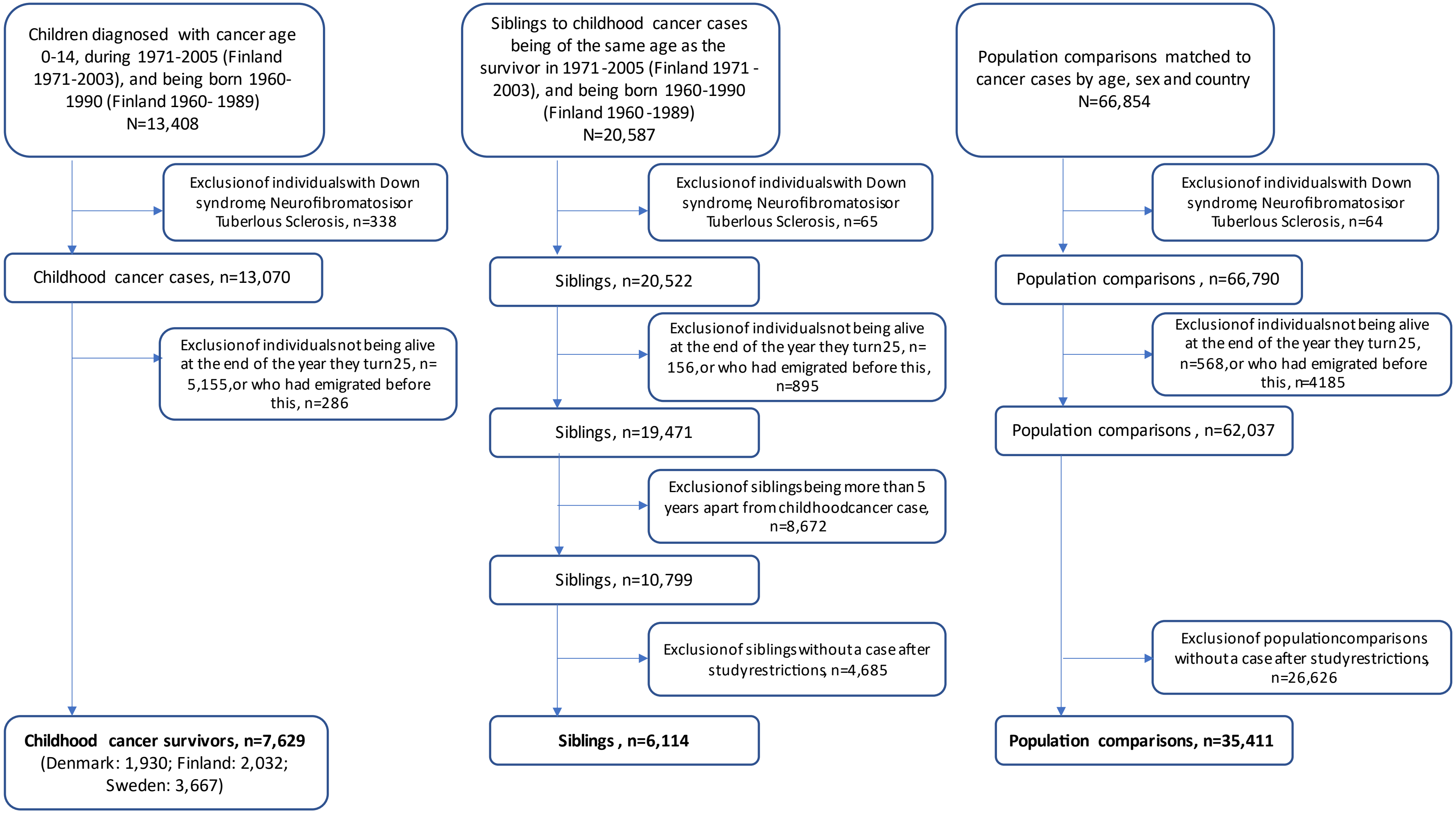

Supplementary Figure 1. Flow chart of the inclusion and exclusion criteria of childhood cancer survivors, population comparisons and siblings.

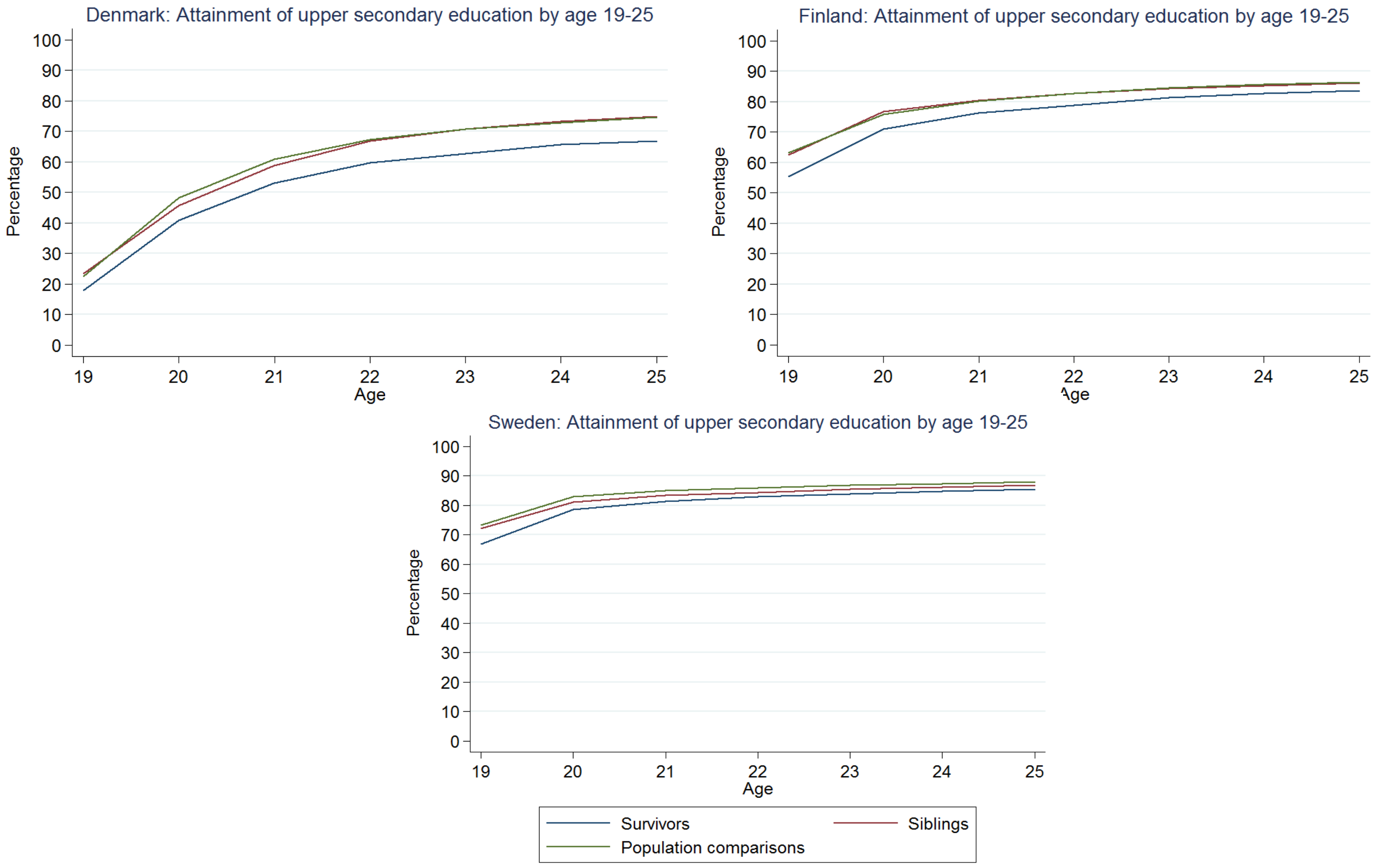

Supplementary Figure 2. Proportion of survivors, population comparisons and siblings having attained upper secondary education by the respective ages 19-25, stratified by country.
